# Biochemical and genetic analyses of N metabolism in maize testcross seedlings: 2. Roots

**DOI:** 10.1007/s00122-018-3071-0

**Published:** 2018-03-14

**Authors:** Ignacio Trucillo Silva, Hari Kishan R. Abbaraju, Lynne P. Fallis, Hongjun Liu, Michael Lee, Kanwarpal S. Dhugga

**Affiliations:** 1 Department of Agronomy, Iowa State University, Ames, IA 50011 USA; 2 Present Address: Global Breeding and Marker Technologies, Dupont Pioneer, 5000 Córdoba, Argentina; 3 Genetic Discovery Group, DuPont Pioneer, Johnston, IA 50131 USA; 4 Present Address: AVX Corporation, One AVX Blvd., Fountain Inn, SC 29644 USA; 5 State Key Laboratory of Crop Biology, Shandong Agricultural University, Tai’an, 271018 China; 6 Present Address: Genetic Resources Program, International Center for Maize and Wheat Improvement (CIMMYT), 56237 El Batan, Texcoco, Mexico

## Abstract

**Key message:**

Intracellular factors differentially affected enzyme activities of N assimilation in the roots of maize testcrosses where alanine aminotransferase and glutamate synthase were the main enzymes regulating the levels of glutamate.

**Abstract:**

N is a key macronutrient for plant growth and development. Breeding maize with improved efficiency in N use could help reduce environmental contamination as well as increase profitability for the farmers. Quantitative trait loci (QTL) mapping of traits related to N metabolism in the root tissue was undertaken in a maize testcross mapping population grown in hydroponic cultures. N concentration was negatively correlated with root and total dry mass. Neither the enzyme activities nor metabolites were appreciably correlated between the root and leaf tissues. Repeatability measures for most of the enzymes were lower than for dry mass. Weak negative correlations between most of the enzymes and dry mass resulted likely from dilution and suggested the presence of excess of enzyme activities for maximal biomass production. Glutamate synthase and alanine aminotransferase each explained more variation in glutamate concentration than either aspartate aminotransferase or asparagine synthetase whereas glutamine synthetase was inconsequential. Twenty-six QTL were identified across all traits. QTL models explained 7–43% of the variance with no significant epistasis between the QTL. Thirteen candidate genes were identified underlying QTL within 1-LOD confidence intervals. All the candidate genes were located in *trans* configuration, unlinked or even on different chromosomes, relative to the known genomic positions of the corresponding structural genes. Our results have implications in improving NUE in maize and other crop plants.

**Electronic supplementary material:**

The online version of this article (10.1007/s00122-018-3071-0) contains supplementary material, which is available to authorized users.

## Introduction

After hydrogen, carbon, and oxygen, nitrogen (N) is the most abundant element in plant tissues. Nearly all of it is derived from the synthetic fertilizers applied to the soil. Variable proportions of the applied soil N are lost to the environment by leaching and denitrification. Leached N flows into the streams and rivers, and eventually into the ocean, supporting algal growth. Excessive algal growth forms “dead zones”, for example, in the Gulf of Mexico, by depleting oxygen in the water, and thus asphyxiating life (Goolsby and Battaglin 2000). Annual delivery of nitrate from the Mississippi river to the Gulf has nearly tripled in the last half century. The size of the dead zone of the Mississippi delta varies depending upon the frequency and the intensity of precipitation in the catchment area of the Mississippi river.

One approach to reduce N loss from the soil is to improve N use efficiency (NUE) of maize. NUE, which in cereals has been defined as the ratio of grain produced per unit of soil N, can be subdivided into two main components: N acquisition efficiency (total plant N/soil N) and N utilization efficiency (total grain yield/total plant N) (Moll et al. 1982; Dhugga and Waines 1989). A comprehensive understanding of N metabolism at the genetic level could provide new avenues to improve NUE in maize (Trucillo Silva et al. 2017).

**Fig. 1:**
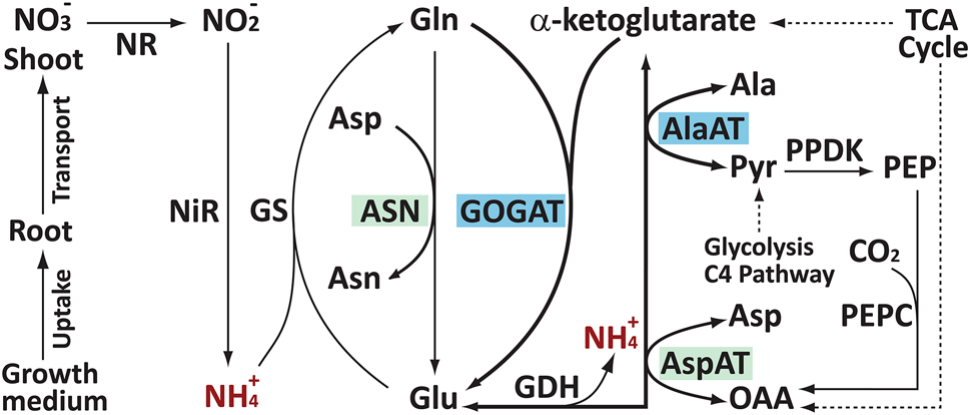
Enzymes and proteins involved in N-acquisition and assimilation in C4 plants (created with Adobe Illustrator CS2). AlaAT and GOGAT each explained more variation in glutamate than AspAT and ASN. *AlaAT* alanine aminotransferase, *ASN* asparagine synthase, *AspAT* aspartate aminotransferase, *GDH* glutamate dehydrogenase, *GOGAT* glutamate synthase, *GS* glutamine synthetase, *NR* nitrate reductase, *NiR* nitrite reductase, *PEPC* phosphoenolpyruvate carboxylase, *PPDK* pyruvate orthophosphate dikinase (adapted from Trucillo Silva et al. 2017)

The model pathway for N reduction and incorporation of reduced N into organic molecules has been well described (Yemm and Folkes 1958; Lea et al. 1990; Lea and Miflin 2010; Plett et al. 2016; Trucillo Silva et al. 2017) (Fig. 1). Nitrate is reduced to nitrite by nitrate reductase (NR) in the cytoplasm, followed by reduction of nitrite in the plastids to ammonium by nitrite reductase (NiR). Ammonium thus generated is coupled to glutamate by glutamine synthetase (GS). Another enzyme, glutamine-2-oxoglutarate aminotransferase (GOGAT) or glutamate synthase, then converts glutamine back to glutamate, producing an additional glutamate from 2-oxoglutarate, thereby initiating the conversion of inorganic N into organic form. This pair of reactions is referred to as GS-GOGAT cycle. Asparagine synthase (ASN) produces asparagine and glutamate from glutamine and aspartate. Glutamate serves as an amino group donor for the formation of other amino acids, a reaction catalyzed by different amino transferases. For instance, alanine aminotransferase (AlaAT) catalyzes the amino group transfer to pyruvate to form 2-oxoglutarate and alanine, while aspartate aminotransferase (AspAT) forms 2-oxoglutarate and aspartate after transferring the amino group of glutamate to oxaloacetate. Following N assimilation, glutamate, asparagine, glutamine and other amino acids are transported via vasculature to the growing organs. Alternatively, they can be stored as vegetative storage proteins, which can aid plant growth during the periods of N deficiency (Dhugga et al. 2007).

Mapping quantitative trait loci (QTL) is a routine in plant genetic investigations and breeding programs. The procedure relies on differences among the trait means of genotypes at a marker locus (Bernardo 2010). The precision in the identification of a QTL is important for the success of further studies, for instance, identification of candidate genes and positional cloning (Remington et al. 2001). That precision in the estimation of the QTL position, referred to as resolution, varies depending on several factors, such as recombination frequency, marker density and population size (Yu et al. 2011).

Much of the current commercial maize germplasm originates from seven progenitor lines, including B73 and Mo17 (Mikel and Dudley 2006). Both inbred lines differ in their response to N fertilization (Balko and Russell 1980) and are parents of the IBM (intermated B73 × Mo17) mapping population (Lee et al. 2002). After ten rounds of random mating, 360 doubled haploid (DH) lines were generated from the IBMSyn10 population (Hussain et al. 2007), which had a higher-resolution for mapping that could be directly associated to the physical map established for the B73 inbred (http://www.maizesequence.org).

Several studies have shown association between QTL and N-metabolism related enzymes (Bertin and Gallais 2001; Hirel et al. 2001; Limami et al. 2002; Zhang et al. 2010, 2015; Liu et al. 2012; Trucillo Silva et al. 2017). We previously described the mapping of various enzymes of N metabolism in the leaf tissue of maize testcrosses (TCs) derived from the IBMSyn10-DH lines and an elite inbred, grown in hydroponics. In this study, we present the mapping of enzymes and metabolites related to N metabolism in the root tissue, and relate those observations to the preceding study of N metabolism in the shoot tissue. This is the first QTL analysis for N-metabolism related enzymes and metabolites in maize roots, particularly in a TC population.

## Materials and methods

### Plant material

From the cross between IBMSyn10-DH lines and an elite inbred (PEI), property of DuPont Pioneer, 176 TC genotypes were generated and used in this investigation. The IBMSyn10-DH population consists of a set of DH lines derived from a population after ten generations of random mating from the cross between B73 × Mo17 (Hussain et al. 2007).

### Experimental design

The same experimental design as described previously was used (Trucillo Silva et al. 2017). Each TC genotype was germinated in autoclaved paper rolls and sterilized water, and subsequently grown under hydroponic conditions. Ten tanks (i.e., sets) containing appropriate growth media were planted with 264 seedlings per tank. In every set, 22 genotypes were grown, and each genotype was replicated 12 times. Two genotypes (B73 and Mo17 each crossed to the PEI) served as controls, and were included in every set and replication.

The growth media consisted of MgSO_4_·7H_2_O 0.5 mM, KH_2_PO_4_ 0.5 mM, Fe-EDTA 0.1 mM, FeEDDHA 0.1 mM, Ca(NO_3_)_2_·4H_2_O 1.25 mM, KNO_3_ 2.5 mM, Na(OH) 0.1 mM, and 0.4 L of trace elements (25 mM H_3_BO_3_, 2 mM MnSO_4_·H_2_O, 2 mM ZnSO_4_·7H_2_O, 0.5 mM CuSO_4_·5H_2_O, 0.5 mM Na_2_MoO_4_·2H_2_O and 50 mM KCl) in a total of 400 L solution per hydroponic tank. The pH was maintained between 5.9 and 6.1 as described in Garnett et al. (2013). A flux density at the canopy level of ~ 500 µmol m^−2^ s^−1^ was supplied at 14 h (25 °C) day: 10 h (20 °C) night cycle. The plants were randomized in the tank every 5 days to guard against the position effects. Two weeks after planting, the six most representative uniform plants of each genotype (based on root and shoot development), were selected and transplanted into another hydroponic tank with same media.

When plants reached V4 stage (Abendroth et al. 2011), usually 4–5 weeks from planting, 4–5 cm of the primary root from six plants were collected and stored at − 80 °C while the rest of the plant tissues were dried for 12 days at 48 °C.

The V4 stage of development was selected for assays because inter-plant shading became a factor after this. The potential border-row effect was mitigated by randomizing the plants more than once during their growth (Tuberosa et al. 2002).

### Biochemical assays

All the biochemical assays were performed as previously described (Trucillo Silva et al. 2017). Activity of eight enzymes related with the N-metabolism pathway was determined in root samples of each genotype. The set of enzymes included NR, NiR, GS, GOGAT, AlaAT, ASN, AspAT and PEPC, and specific protocols were adapted by K. Dhugga, R. Abbaraju and L. Fallis and described in Plett et al. (2016). GS, GOGAT, Asp AT and PEPC assay protocols were adapted from Gibon et al. (2004), NR from Lea et al. (1990), NiR from Bourne and Miflin (1973), ASN from Joy and Ireland (1990), and AlaAT protocol was modified from Ashton et al. (1990). Metabolites nitrate and glutamate were measured as byproducts of enzyme reactions. All measurements were determined by the absorbance of each biochemical reaction compared to known standards using a spectrophotometer (Spectramax Plus 384 Microplate Reader, Molecular Devices).

Plant tissues were weighed and analyzed for N content by combustion analysis as described by DeBruin et al. (2013). Based on root biomass dry weight (RW) and percentage of N measurements (N_r_), total amount of N present in root (TN_r_) tissues was calculated. In addition, N_ratio_ was estimated as the ratio between total amount of N present in shoot tissues (TN_s_) and TN_r_. The analysis of shoot dry weight (SW), TN_s_ and N_s_ is presented in Trucillo Silva et al. (2017).

### Trait data analysis

Statistical analysis was implemented in R statistical program (RCoreTeam 2014) as described in Trucillo Silva et al. (2017). Ggplot2 (Wickham 2010) and GGally (Schloerke et al. 2014) R packages were used for initial analysis of the raw data. First, a univariate analysis, where a single variable is fitted in a model, followed by a multivariate approach, where multiple variables are analyzed simultaneously, was performed to comprehend the relationship among the variables. Then, based on a jackknife resampling strategy, outliers in the dataset were identified as described in Trucillo Silva et al. (2016). The main procedure consists on fitting a statistical model *n* times, systematically omitting one observation from the dataset, followed by the prediction of random effects for a subset of the most consistent genotypes each of the *n* times. The mixed model was fitted with ASReml R package (Butler et al. 2007) and correspondent mixed model equations were solved for the prediction of random effects and estimation of fixed effects.

The statistical model can be represented as follows:

$$y = Xb + Zu + e,$$

where $y$ denotes a *n* × 1 vector of observed response values, $b$ is a *p* × 1 vector of fixed effects, $X$ is a *n* × *p* design matrix, $u$ is a *q* × 1 vector of random effects,$Z$ is a *n* × *q* design matrix, and $e$ being the error term.

The following assumptions were used: *E* (*u*) = 0, *E* (*e*) = 0, Cov (*u*, *e*) = 0, and Var (*u*) = *G* and, Var (*e*) = *R*. The *G* matrix had a compound symmetry structure on the genotype levels and *R* matrix is a diagonal matrix with different values for each set, allowing non-constant variance across sets. The response variable was the activity of the enzyme and the metabolite concentration, respectively. Set, the light replicate and plate were included as fixed effects in the model (where replicate and plate are nested in a set), and the check genotype effect was included as a continuous covariate. Finally, the genotype was included as a random effect in the linear model. Several genotypes were discarded depending on the trait (e.g., for both AlaAT and NR five genotypes were removed). Furthermore, one and four complete sets of data were removed for glutamate and nitrate, respectively, due to the contamination of samples and very low accuracy in the estimations.

Significance of genetic variance was calculated based on log-likelihood ratio test by comparing models with and without the TC random effect. Correlations were calculated among BLUP values for each pair of traits and significance was adjusted after the Bonferroni correction for multiple comparisons. Repeatability was derived from variance estimations from ASReml. The variance components were estimated for each different set. As a result, different values of repeatabilities were estimated and partial estimates were averaged. Path coefficient was performed as described by Wright (1921) and Trucillo Silva et al. (2017).

The studied traits followed Gaussian distribution as judged from the similarity of mean and median values along with skewness estimates (Supplementary Material 1).

### Genotypic information and genetic maps

TC materials were genotyped with 5306 single nucleotide polymorphism (SNP) markers by the Beijing Genomics Institute. Physical and genetic positions of the markers were determined and genetic maps were created with R/qtl (Broman et al. 2003). Recombination fractions were estimated and the Kosambi mapping function was implemented to calculate genetic map distances (Kosambi 1944). In addition, mapping distances were adjusted to compare the results with previous investigations. The expansion factor was determined based on the following equation: $\propto\, = \frac{j}{2} + (2{\text{i}} - 1)/2{\text{i,}}$ where *j* corresponds to the number of generations of intermating including the two generations for generating the F_2_, and *i* is the number of inbred generations after intermating (Teuscher et al. 2005).

The real map was 11,265.25 cM and map distances were reduced by a factor of 6.5 to estimate the adjusted F_2_ map. The final adjusted map was 1733.12 cM length with an average spacing between markers of 0.33 cM, while the maximal spacing between markers was nearly 7 cM, on chromosome 6. With regard to physical distance, the length of the total genome was 2051.75 Mb, with the biggest gap between markers of 69.80 Mb length (located on chromosome 2). On average there was a marker positioned every 400 Kb.

### QTL mapping and identification of candidate genes

QTL Cartographer (Basten et al. 2002) was utilized to detect associations between phenotypes and genotypes. Single-marker analysis, linear regression analysis and composite interval mapping (CIM) was implemented. Zmap (model 6) was performed for CIM, using the ten most significant marker cofactors identified by forward and backward regression. QTL were scanned at intervals of 1 cM and at every marker while cofactors located within a window of 10 cM of the scanned position were excluded from the analysis. To determine LOD score thresholds of 5%, and significant QTL, 1000 permutations were performed for every trait. Two nearby QTL were considered as different when LOD peaks were localized 20 cM or greater apart. Effects of QTL are expressed relative to the B73 allele, where an effect with a positive sign represents an increasing allele from B73 and the one with a negative sign denotes an increasing allele from Mo17.

Multiple interval mapping (MIM) analysis was performed by fitting previously identified QTL from CIM analysis, and parameters were re-estimated and positions refined. All pairwise interactions between QTL in every model were examined for each trait. The significance was determined based on the information criterion: IC (*k*) = − 2 (log (*L*) − *kc* (*n*)/2), where the penalty function corresponds to: *c* (*n*) = log (*n*) and a threshold of 0.0 was used (Basten et al. 2002). The proportion of the total phenotypic variance associated with each model was estimated.

Candidate genes annotated on corresponding 1-LOD QTL confidence interval regions were examined from MaizeGDB (Lawrence et al. 2008) and Phytozome (Goodstein et al. 2012). Those candidate genes directly related to N-metabolism based on descriptions in model species, such as rice (*Oryza sativa*) and Arabidopsis (*Arabidopsis thaliana*), were proposed for further studies. Several other candidate genes may be promising candidates for further investigations, including transcription factors; however, they were not considered due to the difficulties to ascertain a direct relationship with N-metabolism in maize based on available descriptions.

## Results

### Plant dry mass and its relationship to biochemical traits

Shoot and root dry mass, respectively, explained 83 and 17% variation in total plant biomass as determined by path coefficient analysis, which mirrors the actual, average proportion of the two components of the plant across all the TC at 86 and 14% (data not shown). The coefficient of variation (CV) was, respectively, 26.2, 19.3, and 19.9% for the root, shoot, and total biomass. A relatively lower CV of 13.4% for the root/shoot ratio implies that partitioning of dry matter between these two plant parts was less variable than the total dry matter accumulation itself.

**Fig. 2:**
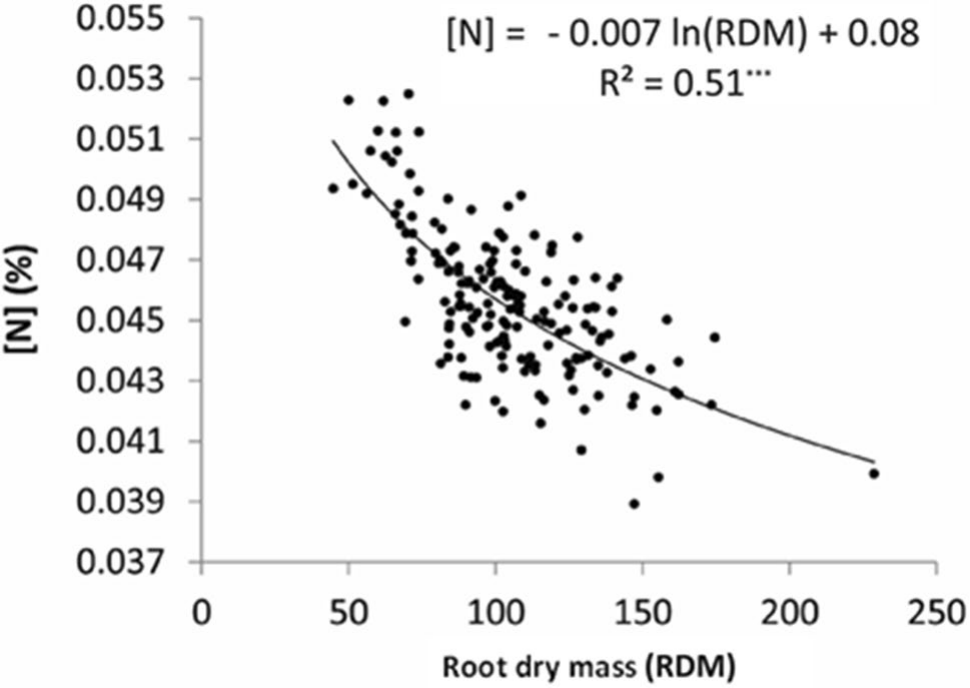
Relationship of root dry mass to root N concentration (created with Adobe Illustrator CS2). The *R*^2^ values for root N versus  shoot dry mass and root N versus total dry mass were 0.35 and 0.39, respectively; *** indicates statistically significant at *P* < 0.001

Root dry mass exhibited a negative, logarithmic relationship with N concentration, with an *R*^2^ of 0.51 (Fig. 2). Shoot N concentration did not correlate with shoot or total dry mass (data not shown). Root N concentration in fact was also negatively correlated with the shoot dry mass and total dry mass with *R*^2^ values of 0.35 and 0.39, respectively, which is not surprising because shoot/root ratio was less variable than either of these traits as discussed earlier. This implies that the roots of the rapidly growing plants retained less N, pointing to a limitation of sink in the shoot for the absorbed N. Apparently, N acquisition, unlike dry matter formation, was not a limiting factor in plant growth. A limitation in dry matter deposition seemingly limited dilution of N in the dry matter, which is manifested in a negative correlation between these two traits. These results are from the plants grown under non-limiting N levels, however, where the root surface was continuously bathed with nitrate. N uptake could become limiting, particularly under low soil N, where a depletion zone develops around the root, particularly during the peak period of transpiration.

**Fig. 3:**
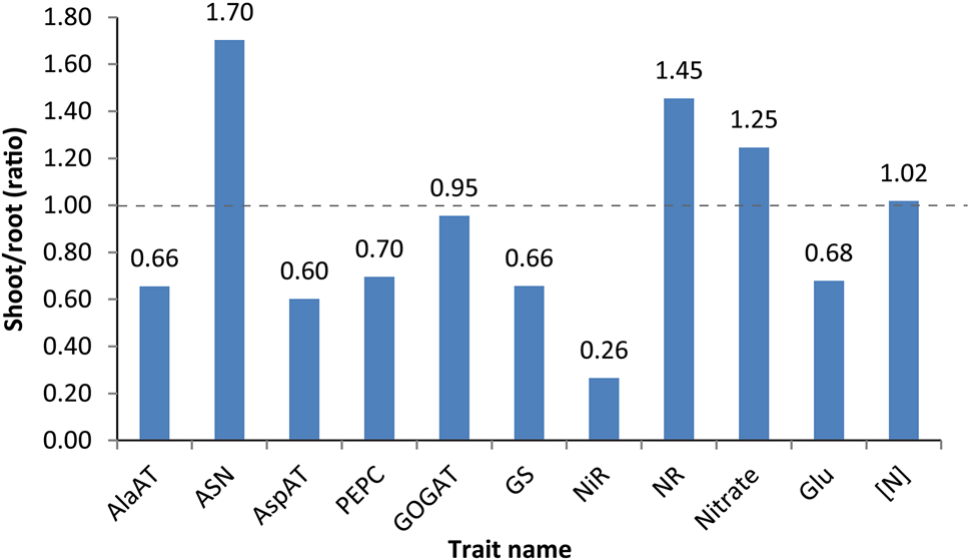
Shoot to root ratios for various enzymes and metabolites in maize IBMsyn10-DH test crosses (created with Adobe Illustrator CS2). The ratio for shoot dry mass to root dry mass was 6.3

ASN and NR had higher activities on a protein basis in the leaves than in the roots, GOGAT was similar between these two tissues, but the activities of the remaining enzymes were higher in the roots at varying levels with NiR being approximately four-fold more active (Fig. 3). Nitrate concentration on dry mass basis was higher in the shoot, implying its efficient transport from the root to the shoot tissue. Glu was more abundant in the roots than in the leaves, suggesting significant nitrate reduction in the roots (Fig. 3).

**Fig. 4:**
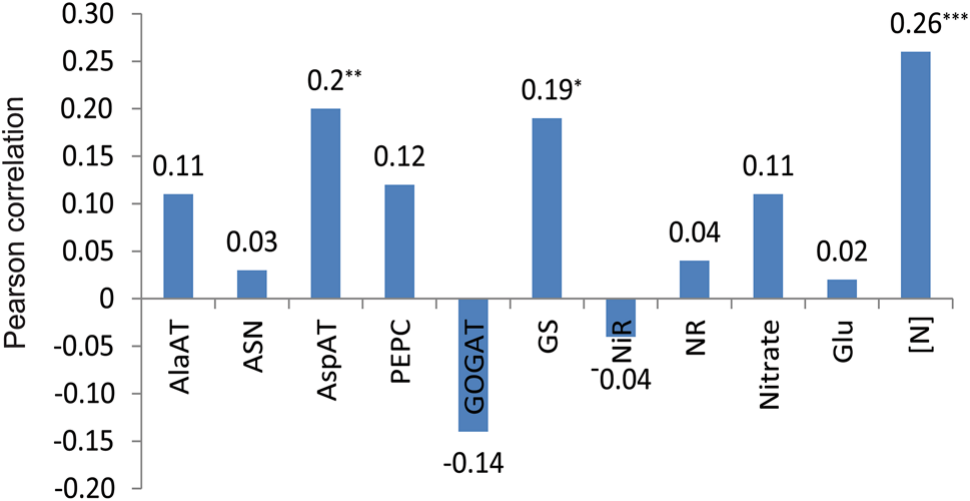
Pearson correlation coefficients for enzyme activities and metabolite concentrations between root and shoot tissues in the IBMSyn10-DH testcross population (created with Adobe Illustrator CS2). *, **, *** indicate statistically significant at *P* < 0.05, 0.01, and 0.001, respectively

Enzyme activities and metabolite concentrations were poorly correlated between the root and the shoot tissues (Fig. 4). The highest correlation coefficient, 0.26, was observed for tissue N concentration. These results imply that the enzymes for N metabolism operate independently in the root and shoot tissues. It is possible that the leaf enzymes are primarily reflective of dry matter deposition, and root enzymes of maintaining N absorption and transport; the relative proportions of these two components in the plant are 84 and 16%, respectively. Furthermore, the reduction of nitrate in the roots may be a mechanism to maintain a favorable electrochemical potential gradient for its continued uptake.

Regression of root and shoot dry matter on each of the enzyme activities revealed a weakly negative relationship for all but two of the enzymes (Table 1). GS explained the most variation, 9%, whereas AspAT and NR each explained approximately 4% of the variation in root dry mass. For the shoot dry mass, AspAT, ASN, GOGAT, NiR, GS, and AlaAT explained 9.5, 7.5, 6.6, 4.7, 4.6, and 3.3% variation, respectively. Of these, AspAT, NiR, and ASN subsumed the variation explained by GOGAT, GS, and AlaAT as indicated by the direct and indirect contributions of each of the enzymes (Trucillo Silva et al. 2017). These observations point to the dilution effect of cell expansion on the cellular contents and suggest that excess capacity for enzyme activities is reflective of metabolic homeostasis to maintain biomass productivity.

**Table 1:** Regression of root and shoot dry mass on enzyme activities for N metabolism in maize testcrosses

Enzyme	Root			Shoot		
	*b*	Intercept	RSQ	*b*	Intercept	RSQ
AlaAT	− 0.051	110.9	0.003	− 0.051	103.5	0.033*
ASN	− 0.033	107.3	0.008	− 0.030	100.3	0.075***
AspAT	− 0.070	109.5	0.042**	− 0.032	101.4	0.095***
GOGAT	0.029	106.7	0.002	− 0.036	100.7	0.066***
GS	− 0.135	117.3	0.087***	− 0.023	102.2	0.046**
NiR	− 0.017	98.4	0.001	0.245	66.4	0.047*
PEPC	0.067	97.2	0.006	− 0.005	103.4	0.000
NR	− 0.641	131.1	0.038*	0.094	87.4	0.005

Variation explained by each enzyme in total dry mass is shown in the RSQ (*R*^2^) column; *, **, *** indicate statistically significant at *P* < 0.05, 0.01, and 0.001, respectively

### Repeatability of N-metabolism associated traits

**Fig. 5:**
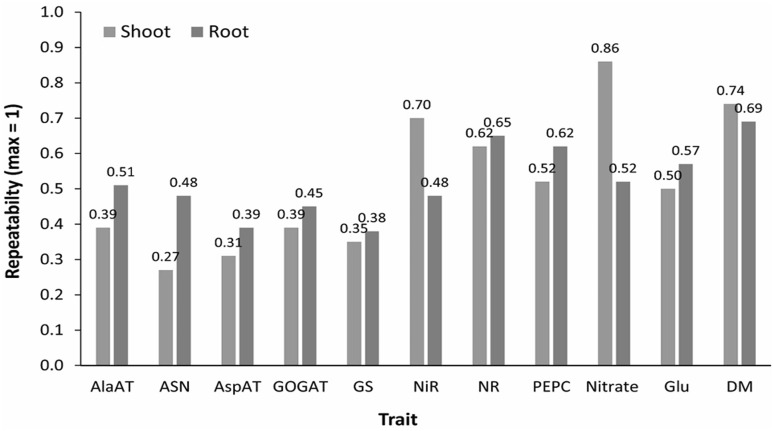
Repeatability values for traits measured on root and shoot tissues in the maize IBMSyn10-DH TC population (created with Adobe Illustrator CS2). *AlaAT* alanine aminotransferase, *ASN* asparagine synthase, *AspAT* aspartate synthase, *GOGAT* glutamine oxoglutarate aminotransferase or glutamate synthase, *GS* glutamine synthetase, *NiR* nitrite reductase, *NR* nitrate reductase, *PEPC* phosphoenol pyruvate carboxylase, *Glu* glutamate, *DM* dry matter. For the shoot tissue, enzyme activities were measured on the youngest, fully expanded leaf at V4 stage (Trucillo Silva et al. 2017)

Repeatability, also referred to as broad-sense heritability in forward selection, is a measure of consistency of a trait among the plants (replicates) of the same TC (Fig. 5). All traits were measured on the same plants grown in the same hydroponic culture system under controlled light and temperature (Table 2). Since the standard error of replicated assays from each plant was negligible, repeatability provides a measure of consistency of different traits among plants in each line. Nitrate concentration and ASN in the shoot tissue were the most and least consistent traits, respectively. Nitrite reductase (NiR) in the leaves was the most consistent enzyme although NR was a close second. PEPC was more uniform in the roots than in leaves, which could be because the light-inducible form in the leaves is the main enzyme for C4 photosynthesis and responds to small changes in light availability. The plants were in close proximity to each other and slight interference from variable shading was unavoidable. The root PEPC was perhaps more stable because its role is primarily anaplerotic, that is, to fix CO_2_ released by respiration into oxaloacetate using phosphoenolpyruvate as the other substrate to support amino acid formation (Fig. 1).

**Table 2:** Sample size, mean values for the population and checks, minimum values, maximum values, standard deviation, coefficients of variation, genetic effect *P* values and repeatability values of the traits measured root tissue from the IBMSyn10-DH TC population of maize

Trait	Unit	*n* ^a^	Pop *µ*^b^	B73TC^c^	Mo17TC^d^	Min^e^	Max^f^	SD^g^	CV^h^	*G* effect *P*^i^	Repeatablity^j^
AlaAT	Glu mg^−1^ protein 0.5 h^−1^ (nmol)	171	241.11	293.38	220.65	189.83	318.37	24.78	10.28	1.75E−12	0.51
ASN	Glu mg^−1^ protein 0.5 h^−1^ (nmol)	176	472.65	474.28	476.30	412.90	538.98	20.43	4.32	1.55E−08	0.48
AspAT	Glu mg^−1^ protein 0.5 h^−1^ (nmol)	176	930.63	963.72	929.91	835.90	1070.29	38.97	4.19	6.75E−07	0.39
GOGAT	Glu mg^−1^ protein 0.5 h^−1^ (nmol)	175	182.80	190.14	192.76	146.11	220.28	12.81	7.01	1.98E−08	0.45
GS	GHA mg^−1^ protein 0.5 h^−1^ (nmol)	176	407.11	453.90	348.95	353.72	471.38	22.08	5.42	2.66E−06	0.38
NiR	Nitrite reduced mg^−1^ protein (nmol)	172	699.90	648.80	589.15	623.88	779.67	33.14	4.73	1.12E−08	0.48
NR	Nitrite produced mg^−1^ protein (nmol)	171	2.82	2.78	1.40	0.03	6.81	1.36	48.23	4.40E−11	0.65
PEPC	NAD reduced min-1 mg-1 protein (µmole)	172	394.85	357.26	423.80	320.09	506.06	40.87	10.35	< 1.00E−12	0.62
Nitrate	nmole mg^−1^ protein	157	199.16	245.32	189.28	160.51	236.25	17.26	8.66	2.99E−09	0.52
Glutamate	Glu mg^−1^ protein 0.5 h^−1^ (nmol)	106	194.38	223.96	192.06	166.71	231.09	12.06	6.20	4.33E−09	0.57
TN_r_	mg	176	4.75	7.10	5.10	2.21	9.13	1.09	22.94	7.12E−07	0.70
N_ratio_	Ratio	176	6.70	6.23	6.45	5.91	7.37	0.26	3.88	3.92E−13	0.50
N_r_	mg g^−1^ (%)	176	4.56	4.37	4.47	3.89	5.25	0.24	5.31	7.76E−11	0.61
N_s_	mg g^−1^ (%)	172	4.64	4.46	4.73	4.31	5.00	12.72	17.98	1.21E−08	0.71
SW	mg	176	661.40	979.73	703.93	374.50	1127.99	127.95	19.34	1.00E−15	0.74
RW	mg	176	105.16	171.54	113.77	44.78	228.75	27.57	26.22	1.12E−11	0.69
TW	mg	176	766.56	1152.94	814.96	419.28	1356.74	152.89	19.94	1.14E−08	0.73

Ns and SW values are included for comparison purposes and presented by Trucillo Silva et al. 2017

^a^Population size

^b^Population mean

^c,d^BLUP value for parental genotypes in testcross genotype

^e^Minimum value

^f^Maximum value

^g^Standard deviation

^h^Coefficient of variation (%)

^i^*P* value of the genetic effect

^j^Repeatability; normalized values were multiplied by a factor of 1.131 for AlaAT, AS and AspAT, and by 1.151 for GOGAT

Nitrate concentration was less variable among plants in the leaves than in the roots, which suggests that roots likely respond to feedback from the leaves for free N status, and thus adjust nitrate absorption to maintain homeostasis in the leaves, where most of it is reduced. This could particularly be the case because nitrate concentration was higher in the leaves than in the roots (Fig. 3).

Despite wide variation in repeatability among the enzymes of N assimilation, both root and shoot biomass were more uniform among the plants sampled for analysis (Fig. 5). Since the plants were selected for uniformity by rejecting the outliers (see Materials and Methods), the root and shoot dry masses are relatively uniform.

### Enzymes for glutamate formation and utilization

**Fig. 6:**
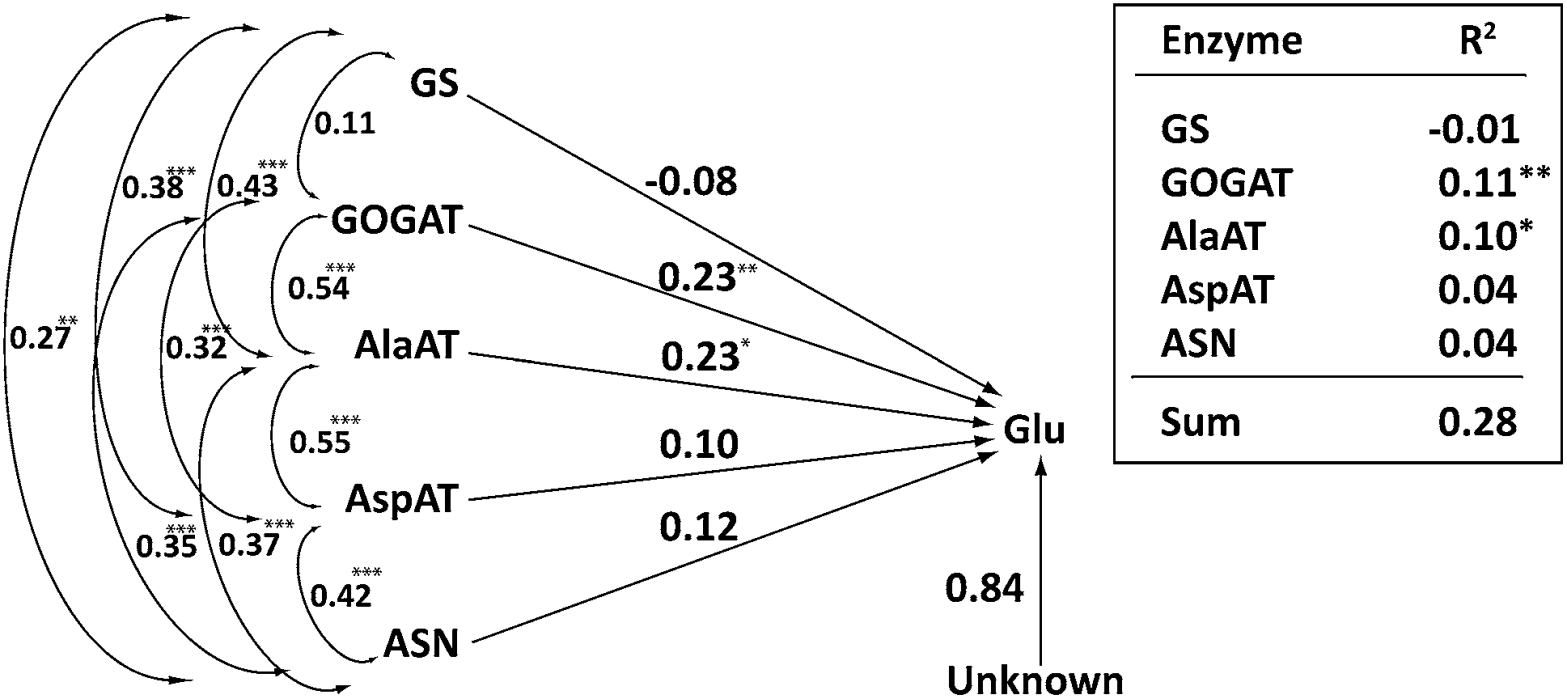
Direct and indirect relationship between glutamate and enzymes directly involved in its formation and utilization (created with Adobe Illustrator CS2). See pathway in Fig. 1. On the right is the table showing partial *R*^2^ for each of the enzymes. Double arrows describe the correlation coefficients between various pairs of enzymes. Path coefficients from each of the enzymes are shown as lines with single arrows. Unexplained path coefficient, which is the square root of the unexplained variation (1 − *R*^2^), is shown separately. *, **, *** indicate statistically significant at *P* < 0.05, 0.01, and 0.001, respectively

Glutamate is a key amino acid in N assimilation as it constitutes the entry step of inorganic N into organic form (Trucillo Silva et al. 2017). A path coefficient diagram depicting the direct and indirect effects of various enzymes toward the cellular glutamate concentration is shown in Fig. 6. In the leaf tissue, AlaAT alone explained 58% of the variation in glutamate, suggesting a key role in maintaining the intracellular concentration of this amino acid (Trucillo Silva et al. 2017). No single enzyme was as strongly correlated with glutamate in the roots, however, perhaps because roots act more as conduits for nitrate uptake but not for primary N assimilation. AlaAT alone still explained 10% of the net variation in glutamate level, but GOGAT was just as important, explaining 11% of the variation (Fig. 6). AspAT and ASN each explained 4% variation in the glutamate level. GS was inconsequential in determining glutamate level, which implies that glutamine, a substrate for glutamate regeneration along with α-ketoglutarate (Fig. 1), was not a limiting factor in the root-cell glutamate level (Fig. 6). A limitation of carbon skeletons because of their utilization in dry matter deposition in expanding cells might favor N cycling between glutamate and alanine (C/N ratio 3) than between glutamate and aspartate (C/N ratio 4) (Trucillo Silva et al. 2017). This pattern appears less prominent in the roots, perhaps because they are not the primary sites of carbon fixation, N reduction and assimilation (Fig. 6). Pairwise correlation coefficients for all the traits are shown in Supplementary Material 2. Of all the pairwise correlations, 31% were highly significant (*p* value < 0.001). All significant correlations between enzyme activities, enzymes and metabolites, and between metabolites were positive.

### Identification of quantitative trait loci

#### Composite interval mapping

**Fig. 7:**
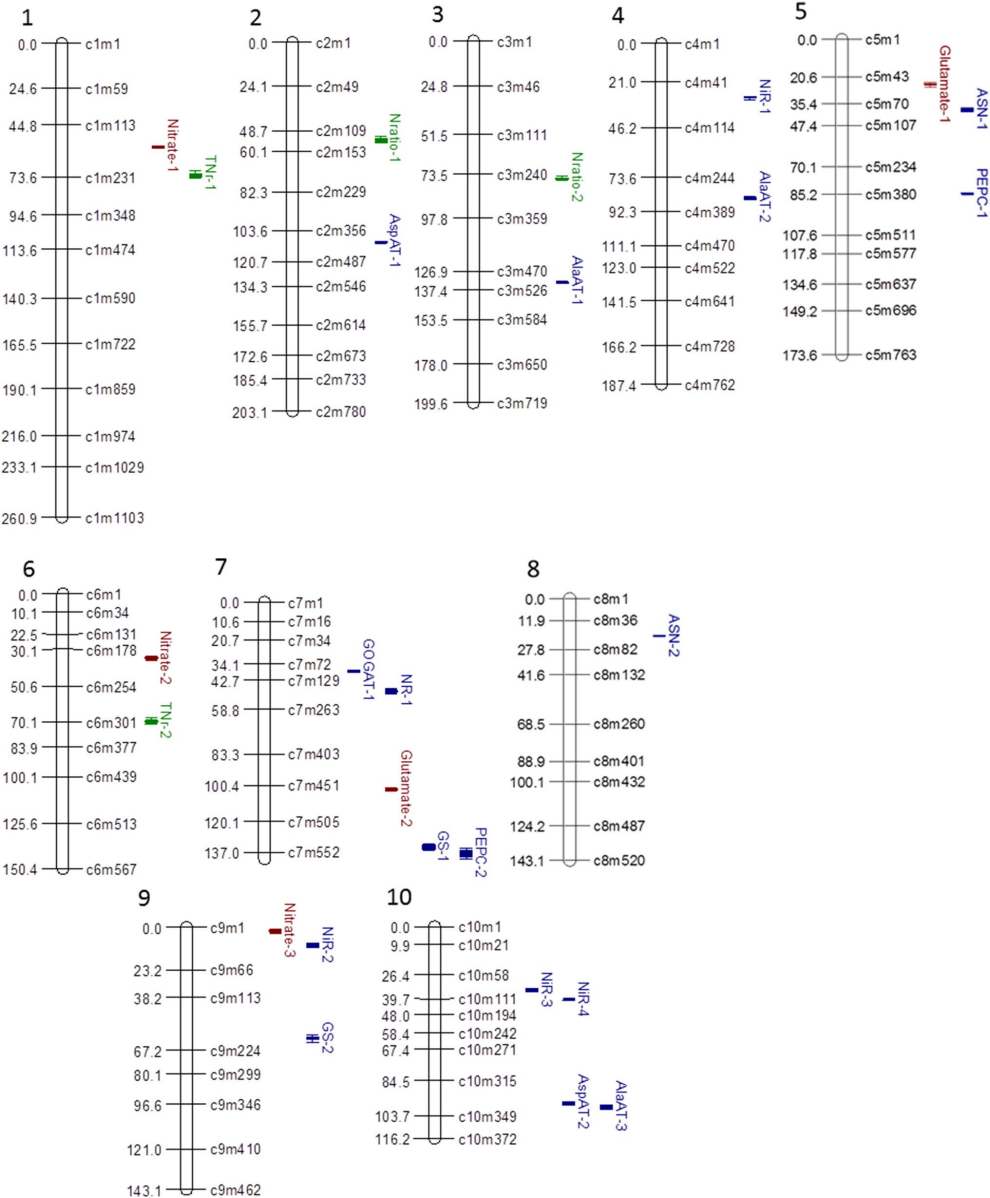
Genetic map and distribution of QTL associated with N metabolism related enzymes and metabolites measured on root tissue in the maize IBMSyn10-DH TC population. Created with MapChart 2.2 (Voorrips 2002). QTL positions are shown at left of chromosomes (in cM) and the lengths of  QTL bars are determined by 2-LOD confidence intervals. Only selected markers are displayed in the figure to the right of chromosomes. QTL associated with the enzyme activities are in blue, while QTL associated with metabolites are in red (color figure online)

Twenty-six QTL were identified across all the traits. Five QTL were detected on one chromosome, 7, whereas only one was identified on chromosome 8 (Fig. 7). AlaAT-3 and AspAT-2 were the only QTL that overlapped their respective 1-LOD CI on chromosome 10. The number of QTL varied for different traits, ranging from only one for some traits (GOGAT and NR) to four for NiR (Fig. 7).

A majority of the QTL, 19, explained less than 10% of the genetic variance. Six QTL explained 10–25%, while one explained > 25% of the variance. The QTL which accounted for the highest amount of variance (31.5%) and presented the highest LOD score (23.4) was for PEPC-1, located on chromosome 5. For that QTL, the B73 allele had a negative effect (− 23.78 µmole NADH/min/mg protein). Furthermore, for 70% of all QTL detected across traits, B73 alleles had negative additive effects. For certain traits, for example, AspAT, GOGAT, and GS, B73 alleles exhibited only a negative effect, however, for ASN QTL, B73 alleles had positive effects (Table 3).

**Table 3:** QTL associated with N metabolism related enzymes and metabolites from root tissue in the IBMSyn10-DH TC maize population

QTL name	Chr^a^	Marker^b^	*G* Pos (cM)^c^	*G* interval (cM)^d^	Adj (cM)^e^	*P* Pos (Mb)^f^	*P* interval (Mb)^g^	LOD	*r*^2^ (%)	Add effect^h^	# Genes^i^
AlaAT-1	3	429	867.44	865.09–868.13	133.45	193.90	192.00–194.20	4.33	7.16	−10.27	84
AlaAT-2	4	264	555.33	552.56–557.69	85.44	146.05	143.75–146.05	4.93	8.14	7.76	63
AlaAT-3	10	292	643.80	641.20–648.66	99.05	146.15	146.05–146.25	4.89	8.06	−7.41	17
ASN-1	5	79	249.44	246.16–258.51	38.38	9.55	9.00–9.85	5.52	8.55	6.27	41
ASN-2	8	45	131.64	131.16–132.20	20.25	6.85	6.85–7.15	4.66	7.16	6.13	6
AspAT-1	2	339	715.00	714.43–718.51	110.00	168.55	165.65–169.35	7.32	11.18	−17.10	115
AspAT-2	10	288	627.89	625.33–631.09	96.60	145.45	145.25–145.45	6.37	9.21	−12.41	6
GOGAT-1	7	84	245.75	241.98–248.84	37.81	13.85	13.85–14.10	6.22	9.77	−4.53	11
GS-1	7	409	873.64	865.44–880.88	134.41	174.25	173.65–174.25	4.25	6.85	−6.17	37
GS-2	9	164	393.47	388.70–398.86	60.53	27.05	26.65–28.30	4.35	7.02	−6.28	63
NiR-1	4	55	191.89	189.18–194.26	29.52	7.25	6.75–7.25	6.76	10.61	11.86	24
NiR-2	9	29	61.20	57.89–70.10	9.42	3.75	3.65–4.65	6.28	9.81	11.10	29
NiR-3	10	77	230.71	221.50–233.11	35.49	10.55	10.45–10.85	5.69	10.57	−13.59	18
NiR-4	10	101	262.07	260.48–263.82	40.32	17.80	15.95–19.05	7.67	12.21	16.93	96
NR-1	7	136	315.63	313.05–321.94	48.56	80.75	78.95–100.05	5.49	9.11	−0.43	376
PEPC-1	5	298	551.15	548.52–552.56	84.79	138.25	127.35–139.25	23.40	31.54	−23.78	218
PEPC-2	7	409	887.11	882.06–906.52	136.48	174.70	174.55–175.55	4.36	4.48	−8.95	60
Glutamate-1	5	50	159.48	157.65–162.73	24.54	5.65	5.45–5.65	8.76	15.00	−6.84	17
Glutamate-2	7	327	666.28	663.82–668.75	102.50	163.55	163.50–164.05	5.75	9.09	−5.43	33
Nitrate-1	1	127	370.69	369.37–372.41	57.03	23.45	23.45–23.80	6.99	14.01	−5.37	12
Nitrate-2	6	155	227.31	223.79–231.96	34.97	94.60	93.55–94.85	5.04	9.48	−4.10	48
Nitrate-3	9	11	12.29	6.99–16.15	1.89	1.55	1.25–1.60	4.79	9.13	4.12	12
TN_r_-1	1	195	469.95	467.09–480.29	72.30	37.95	37.15–39.35	4.38	6.04	−0.28	79
TN_r_-2	6	235	451.99	449.76–457.93	69.54	115.45	115.10–117.50	4.54	6.27	0.29	100
N_ratio_-1	2	119	349.98	344.92–359.17	53.84	16.25	15.85–16.65	4.37	6.39	−0.07	40
N_ratio_-2	3	212	493.48	490.50–497.62	75.92	133.50	129.25–136.95	7.03	10.68	−0.09	185

^a^Chromosome number

^b^Marker localized at LOD peak

^c^Genetic position of molecular marker in cM

^d^1-LOD interval in cM

^e^Adjusted genetic position

^f^Physical position in Mb

^g^1-LOD physical interval

^h^Additive effect of QTL (a positive-signed effect represents an increasing allele from B73, while a negative-signed allele denotes an increasing allele from Mo17)

^i^Number of annotated genes underlying 1-LOD QTL confidence interval

Confidence intervals (CI 1-LOD) for QTL ranged from 1.04 to 24.46 cM (0.16–3.76 cM adjusted distance) length, with an average of 7.79 cM (1.2 cM adjusted distance). Those CI correspond to 0.2–21.1 Mb in physical distance, with a mean CI length of 2.46 Mb (Table 3).

#### Multiple interval mapping

First order epistatic interactions between QTL identified previously by CIM were not significant for any of the traits, thus epistatic digenic effects were excluded from genetic models. Even though 43% of the total variance was explained in PEPC by fitting two QTL in an MIM model, other genetic models captured less than 10% of the phenotypic variance, such as for ASN, GOGAT, GS and NR (Table 4). On average, multiple QTL models explained 15.1% of the variance when two QTL were included in each of the models.

**Table 4:** Analysis of multiple QTL models for N metabolism related enzymes and metabolites measured on root tissue from the maize IBMSyn10-DH TC population

Phenotype	# QTL in model^a^	Model *R*^2^ (%)^b^
AlaAT	2	11.65
ASN	2	8.07
AspAT	2	12.06
GOGAT	1	9.77
GS	2	6.59
NiR	4	26.42
NR	1	9.11
PEPC	2	42.53
Nitrate	3	15.77
Glutamate	2	18.86
TN_r_	2	8.12
N_ratio_	2	12.85

^a^Number of QTL fitted in MIM model

^b^Total *R*^2^ obtained by fitting significant QTL simultaneously in a MIM model

#### Candidate genes

An average of 63 genes were annotated underlying QTL 1-LOD regions, with CI regions containing between six and 376 genes. Only a subset of the putative genes could be associated to N-metabolism pathway based on their descriptions in model species. The most promising genes were GRMZM2G028574, GRMZM2G111225, GRMZM2G136712, GRMZM2G155974, GRMZM2G166366, GRMZM2G374302, GRMZM2G409131, GRMZM2G466543, GRMZM2G473001, GRMZM2G481529, GRMZM5G817058, GRMZM2G575696 and GRMZM2G580894 (Table 5). Each of them was associated with a putative function relevant to NUE. Examples are PEPC, nitrilase, aspartate kinase, glutathione synthetase, aspartate kinase, arginine decarboxylase, phosphofructokinase, arogenate dehydratase, phosphopyruvate hydratase, phosphoribosyl transferase, and last two genes as *S*-adenosyl-methionine-dependent (SAM)-methyltransferase, respectively. In agreement with our earlier study on the leaves of this population (Trucillo Silva et al. 2017), all the QTL identified in this study are located on a different position to the known genomic location of each corresponding structural gene. For example, GS QTL were identified on chromosomes 7 and 9 at physical positions 158.15 and 23.85 Mb in this study, whereas GS1 and GS2 locus are located in chromosomes 1, 2, 4, 5, 9 (between 146.06 and 146.07 Mb), and 10, based on the following nearest loci on the IBM2 2008 Neighbors map, respectively. The candidate genes identified within the QTL regions might affect the enzyme activities in a *trans*-acting regulatory manner as previously described, most likely through metabolic pathways as all the genes are non-regulatory in nature (Zhang et al. 2010). The candidate genes we identified are located on chromosomes 1, 2, 3, 4, 6, and 7. No candidate genes related to N metabolism were identified underlying QTL for ASN, GS, NiR, PEPC, nitrate, and glutamate.

**Table 5:** Candidate genes underlying 1-LOD QTL regions associated with N metabolism related enzymes and metabolites measured on root tissue from the maize IBMSyn10-DH TC population

Maize GDB ID	Corresponding gene annotation	Chr^a^	Start^b^	End^c^	QTL name
GRMZM2G028574	PEPC 3	6	115914515	115915086	TN_r_-2
GRMZM2G111225	Nitrilase 2	4	145590144	145596571	AlaAT-2
GRMZM2G136712	Aspartate kinase	7	80189428	80201455	NR-1
GRMZM2G155974	Glutathione synthetase	3	133812995	133826187	N_ratio_-2
GRMZM2G166366	Aspartate kinase	6	115555315	115557026	TN_r_-2
GRMZM2G374302	Arginine decarboxylase	4	144862958	144868207	AlaAT-2
GRMZM2G409131	Phosphofructokinase	7	82344751	82349620	NR-1
GRMZM2G466543	Arogenate dehydratase 6	2	166506882	166509171	AspAT-1
GRMZM2G473001	PEPC 1	7	86459173	86464913	NR-1
GRMZM2G481529	Phosphopyruvate hydratase	1	38637579	38641262	TN_r_-1
GRMZM5G817058	Phosphoribosyl transferase	7	80946776	80947644	NR-1
GRMZM2G575696	SAM-methyltransferase	7	85199074	85200388	NR-1
GRMZM2G580894	SAM-methyltransferase	7	83464904	8347015	NR-1

^a^Chromosome

^b,c^Start and end location in bp

## Discussion

Previously, we reported the genetic and biochemical analyses of shoot enzymes and metabolites in a TC mapping population (Trucillo Silva et al. 2017). In this report, we focus on the root tissue of the same population.

Variable repeatabilities for various enzymes and metabolites both for root and shoot tissues provide a window into the stability of each of these traits within a line (Fig. 5). Despite the fact that a controlled environment was used (light intensity, nutrients, and temperature) in which the testcrosses were grown to the maximum extent possible, repeatability for a majority of the biochemical traits was generally less than 50% both in the roots as well as in the leaves (Fig. 5). Higher repeatability for the root and shoot dry mass than most of the enzymes suggests that enzyme levels can fluctuate to maintain metabolic homeostasis such that dry matter deposition is maintained. A slight negative correlation between dry mass and enzyme activities suggests that the enzyme amounts are maintained above the threshold for optimal biomass production (Table 1). Nevertheless, variation for repeatability highlights how a trait can vary significantly among plants of the same genotype grown in a controlled environment. These observations are significant in that they imply that environmental variation may be difficult to control under the field conditions regardless of measures taken. Increasing the number of replications and years or locations for testing genetic variants could help reduce the chances of false positives.

A lack of correspondence between the enzyme activities of the root and shoot tissues suggests that metabolism in these tissues is optimized for different functions, apparently for dry matter accumulation in the shoot and nutrient absorption and transport in the root.

Even though numerous QTL associated with enzymes involved in N-metabolism were identified in previous studies (Agrama et al. 1999; Limami et al. 2002; Canas et al. 2012), only a few investigations were based on a representative and high-resolution mapping population, such as Zhang et al. (2010) and (2015). The performance of most traits in maize in the inbred lines is weakly, if at all, correlated with their hybrid (Hallauer et al. 2010). Yet, only a few studies have focused on mapping in testcross populations (Bertin and Gallais 2001; Gallais and Hirel 2004). Furthermore, we used TC derived from the IBMsyn10 population, which represent a higher recombination frequency, and thus narrower intervals of the identified QTL. To account for the higher recombination rate, we used a platform with 5303 SNP markers.

As reported in previous studies, the activity of enzymes investigated, constituents of the N-metabolism pathway (except PEPC, which is a member of the primary C-metabolism), seem to be co-regulated (Zhang et al. 2010; Trucillo Silva et al. 2017). Hence, a positive correlation between enzyme activities, as well as within metabolites concentration, was expected and our observations confirmed it. Significant correlations between enzyme activities and metabolites were also positive (Supplementary Material 2).

In comparison to previous studies (Zhang et al. 2010; Trucillo Silva et al. 2017), in which leaf tissue was investigated, determination of root enzyme activity was more prone to sample to sample variation, mainly because, unlike the leaf where the same position could be sampled from each plant, root lengths were more variable, as reflected in final samples. Performance of assays on six replications per genotype ensured that the repeatability measures are quite similar between the root and leaf enzymes (Fig. 5).

A few of the QTL identified in this study were found to be in analogous positions as in previous detected NUE-related QTL on leaf tissues (Trucillo Silva et al. 2017). For instance, a root QTL associated with ASN located on chromosome 5, corresponds to leaf QTL for PEPC, nitrate and GOGAT (LOD peak values identified 2, 4 and 7 adjusted cM apart, respectively). In agreement with Zhang et al. (2010), a QTL for AlaAT was detected on chromosomes 4, about 5 cM away from the detected position in this study. Nonetheless, most of the QTL reported in other maize studies (Agrama et al. 1999; Hirel et al. 2001; Canas et al. 2012), which failed to co-locate, were greater than 20 cM away or even on different chromosomes compared to the QTL identified in this investigation. For example, QTL for GS activity were determined on chromosomes 7 and 9 in this study, whereas on chromosomes 4 and 5 in a previous study (Canas et al. 2012).

A lower number of QTL was identified per trait compared to previous investigations on leaf tissues (Zhang et al. 2010; Trucillo Silva et al. 2017). This suggests that similar traits are differentially regulated in roots and leaf tissues. The power to identify a QTL depends on the magnitude of the QTL effect and the size of the segregating population (Beavis 1998). Because a large number of small-effect QTL segregating in the genome were expected, and due to the size of the segregating population (176 individuals), only a subset of the total number of QTL was expected to be identified. Moreover, in comparison to Zhang et al. (2010), the number of QTL detected were most likely affected by the six additional rounds of random mating before fixing the lines that constituted the IBMsyn10-DH population. It is possible that the QTL previously detected in large linkage blocks, might have been separated into several smaller-effect QTL after further recombination events occurred. Therefore, the power to detect a QTL, each with a very small effect, would be expected to be lower. Another difference between the two studies is the use of inbred versus hybrids for mapping QTL. Little evidence of common QTL detection between inbred per se and TC progeny has been found in previous investigations (Beavis et al. 1994; Schon et al. 1994). It is possible also that some of the QTL identified in the inbred lines could have been masked in heterozygous form.

As previously reported by Trucillo Silva et al. (2017), the MIM results across traits suggest that there might be several undetected small effect QTL responsible for the rest of the genetic variation, for example, for PEPC and ASN, two QTL explained 42.5 and 8.1% of the variance, respectively. The sum of the effect of numerous QTL, each with small marginal effect, plus any type of epistasis which they might be involved in, should account for all the unexplained genetic variance in the MIM QTL models. It has been established that epistasis can make a large contribution to the genetic regulation of complex traits (Carlborg and Haley 2004). However, statistically significant first order epistasis between identified QTL was not detected. Likewise, no significant epistasis between QTL was detected in a recent study based on the maize nested association mapping (NAM) population, which included the parents of this population (B73 and Mo73) (Zhang et al. 2015).

From a total of 60,000 annotated genes across the maize genome, a limited number was identified under 1-LOD QTL intervals. One of the genes, GRMZM2G368398, an oligopeptide transporter, was also identified in a previous meta-QTL investigation of candidate genes for NUE in maize (Liu et al. 2012). An additional gene (GRMZM2G053958), which codes for NAD(P)-binding Rossmann-fold superfamily protein was proposed as a candidate gene in a recent investigation based on C and N metabolism in the NAM population (Zhang et al. 2015). In this study, 13 candidate genes associated with N-metabolism are suggested for further studies. GRMZM2G028574 and GRMZM2G473001 are annotated as PEPC genes. GRMZM2G111225 is annotated as a nitrilase enzyme, which catalyzes the hydrolysis of nitriles to carboxylic acids and ammonia, and is implicated in auxin biosynthesis in maize (Park et al. 2003). GRMZM2G166366 and GRMZM2G136712, code for aspartate kinases, which catalyze the phosphorylation of aspartate to for β-aspartyl phosphate, and is responsible for the first step in the biosynthesis of the amino acids lysine, methionine, and threonine (Azevedo et al. 1992). GRMZM2G155974 catalyzes the addition of glycine to γ-glutamyl-cysteine, generating glutathione. Glutathione is a key water-soluble antioxidant, which represents the storage form and long-distance transport form of reduced sulfur (Zagorchev et al. 2013). GRMZM2G374302 codes for arginine decarboxylase, a key enzyme involved in polyamine biosynthesis that decreases in concentration under N-deficiency conditions (Amiour et al. 2012). In addition, GRMZM2G409131 catalyzes the phosphorylation of d-fructose 6-phosphate to fructose 1,6-biphosphate, the entry point into glycolysis (Plaxton and Podesta 2006). GRMZM2G466543 codes for arogenate dehydratase, a gene that functions in the final steps of the aromatic amino acid pathway that produces two essential amino acids, tyrosine and phenylalanine, which initiate lignin formation, releasing ammonium as a byproduct that is again absorbed by the GS/GOGAT cycle (Holding et al. 2010). GRMZM2G481529 is a cytosolic enolase or phosphopyruvate hydratase and is described as a metalloenzyme responsible for the conversion of 2-phosphoglycerate to PEP, necessary for sucrose synthesis from pyruvate in C4 plants (Karpilov et al. 1978). GRMZM5G817058 is a phosphoribosyltransferase and acts in amino acid metabolism by catalyzing the first step in the biosynthesis of histidine (Morot-Gaudry et al. 2001). Finally, GRMZM2G575696 and GRMZM2G580894, both S-adenosyl-l-methionine (SAM)-dependent methyltransferases, are responsible for transferring methyl groups from the methyl donor SAM to N, oxygen, sulfur, and C atoms of several biomolecules, such as DNA, RNA, histones, and other proteins. These modifications may affect the expression of a wide variety of genes involved in signaling, nuclear division, and metabolism (Bobenchik et al. 2011).

## Conclusions

Enzymes for N metabolism exhibit relatively low repeatabilities as compared to dry mass, suggesting they might be overexpressed in the cells under normal N possibly to maintain biomass accumulation through metabolic homeostasis. Poor or no correlation between the root and leaf enzymes and metabolites signifies the importance of studying these two tissues separately. Mildly negative correlations between dry mass and tissue N concentration as well as between the enzymes and dry mass appear to arise from a lack of dilution of cellular contents because of constraints on cell expansion, which, in turn, might arise from factors other than N that limit dry matter formation. Glutamate synthase and alanine aminotransferase were the key enzymes in regulating the cellular levels of glutamate. Our high-throughput assays pave the way to study the enzymes and metabolites of N utilization at field scale.

### Author contribution statement

KSD, ML, and ITS conceived and designed the experiments, carried out data analyses, and wrote the manuscript. ITS, HKRA, and LPF performed the laboratory analyses, HL provided molecular marker information. All authors revised and approved the final manuscript.

## Electronic supplementary material

Below is the link to the electronic supplementary material.

Mean, median and skewness of the N-metabolism related enzymes and metabolites measured on root tissues in the maize IBMSyn10-DH TC population (created with Adobe Illustrator CS2) (TIFF 104 kb)

Correlation matrix-heatmap of the N-metabolism related enzymes and metabolites measured on root tissues in the maize IBMSyn10-DH TC population (created with corrplot package, R). Significant correlation values (p value <0.05) depicted in blue (positive correlation) and red (negative correlation) (TIFF 1183 kb)
