# Supplementary figures and images for: Biochemical and genetic analyses of N metabolism in maize testcross seedlings: 2. Roots
Source: Theor Appl Genet. 2018 Mar 14;131(6):1191–205. doi: 10.1007/s00122-018-3071-0 (PMC5945762; doi:10.1007/s00122-018-3071-0)

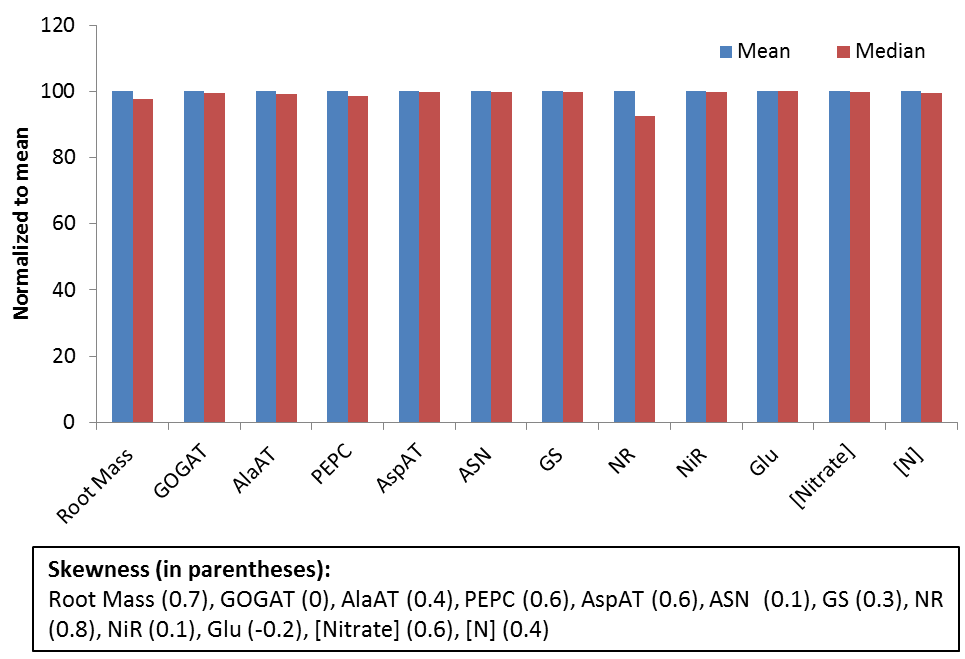

Supplement: Supplementary file 1 — Mean, median and skewness of the N-metabolism related enzymes and metabolites measured on root tissues in the maize IBMSyn10-DH TC population (created with Adobe Illustrator CS2) (TIFF 104 kb) [file 122_2018_3071_MOESM1_ESM.tiff]

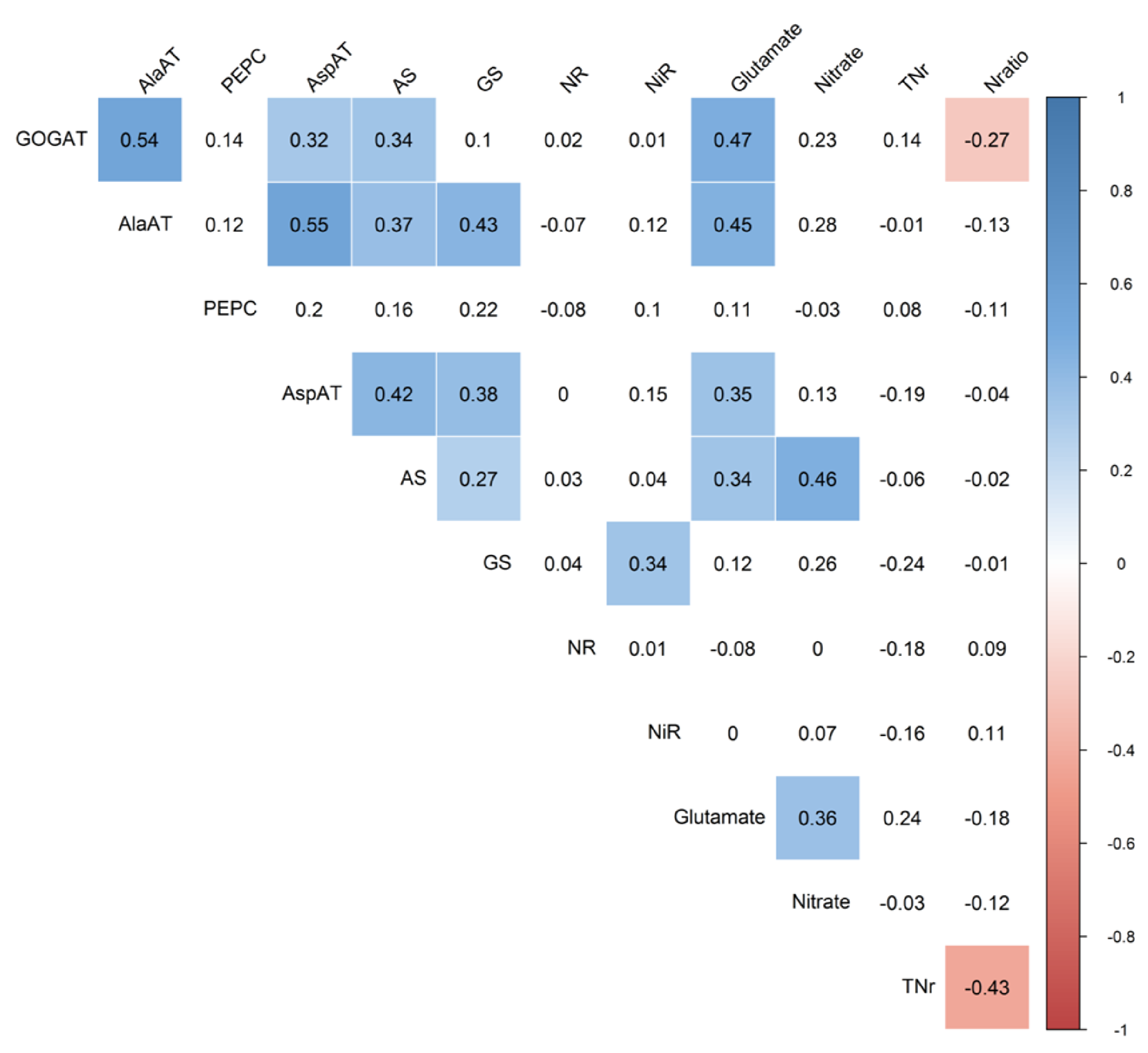

Supplement: Supplementary file 2 — Correlation matrix-heatmap of the N-metabolism related enzymes and metabolites measured on root tissues in the maize IBMSyn10-DH TC population (created with corrplot package, R). Significant correlation values (p value <0.05) depicted in blue (positive correlation) and red (negative correlation) (TIFF 1183 kb) [file 122_2018_3071_MOESM2_ESM.tiff]
